# Ruxolitinib and decitabine plus a busulfan–cyclophosphamide conditioning regimen for relapse prophylaxis in patients with high-risk acute myeloid leukemia or myelodysplastic syndromes

**DOI:** 10.3389/fimmu.2025.1586512

**Published:** 2025-08-18

**Authors:** Yujun Wei, Songhua Luan, Lu Wang, Lili Wang, Fei Li, Xiangshu Jin, Ruoling Yang, Kun Qian, Bo Peng, Jingwen Tang, Haoyang Zhang, Liping Dou, Daihong Liu

**Affiliations:** ^1^ School of Medicine, Nankai University, Tianjin, China; ^2^ The Chief Department of Hematology, the Fifth Medical Center of Chinese People's Liberation Army (PLA) General Hospital, Beijing, China; ^3^ Department of Hematology, the First Medical Center of Chinese People's Liberation Army (PLA) General Hospital, Beijing, China

**Keywords:** acute myeloid leukemia, myelodysplastic syndrome, allogeneic hematopoietic stem cell transplantation, conditioning regimen, relapse

## Abstract

**Purpose:**

Relapse remains the leading cause of treatment failure in high-risk acute myeloid leukemia (AML) or myelodysplastic syndrome-IB (MDS-IB) patients after allogeneic hematopoietic stem cell transplantation (allo-HSCT). Ruxolitinib has demonstrated antileukemic activity *in vitro*, and decitabine has been found to be tolerable when combined with modified busulfan–cyclophosphamide (mBu/Cy) conditioning regimen. Here, we investigated the efficacy of ruxolitinib and decitabine plus a mBu/Cy conditioning regimen (Rux-Dec-mBu/Cy) in reducing relapse in high-risk AML/MDS patients (*ClinicalTrials.gov identifier: NCT04582604*).

**Patients and methods:**

This prospective investigational study enrolled 58 patients between May 2020 and July 2023. These patients had either a relapsed/refractory status, remission status with adverse genetic abnormalities or positive measurable residual disease (MRD+) prior to conditioning. Ruxolitinib (days –15 to –1) and decitabine (days –15 to –10) were administered, followed by mBu/Cy conditioning. The outcomes of a historical cohort of 58 patients (matched 1:1) who received mBu/Cy are described for reference.

**Results:**

All 58 patients achieved engraftment. With a median follow-up of 967 (464–1597) days, the 2-year cumulative incidence of relapse was 19.0%. The probabilities of 2-year overall survival (OS), disease-free survival (DFS) and graft-versus-host disease-free, relapse-free survival (GRFS) were 70.3%, 70.6% and 65.2%, respectively. The cumulative incidence of grade II-IV acute graft-versus-host disease (aGVHD) was 44.1%. The most common grade ≥3 adverse event was oropharyngeal mucositis (8.6%, n=5). Within 6 months post-transplantation, the cumulative incidence of cytomegalovirus (CMV) reactivation was 34.5%, and that of Epstein–Barr virus (EBV) reactivation was 62.1%.

**Conclusions:**

This investigational study revealed that the Rux-Dec-mBu/Cy conditioning was tolerable and reduced relapse in high-risk AML/MDS patients.

## Introduction

Relapse is the major cause of treatment failure, contributing to 40–50% of mortality rates in patients with high-risk acute myeloid leukemia (AML) or myelodysplastic syndrome-IB (MDS-IB) after allogeneic hematopoietic stem cell transplantation (allo-HSCT) (1, 2). Relapsed/refractory status, adverse risk disease features and measurable residual disease (MRD) before allo-SCT increase the risk of relapse and are consistently associated with shortened survival (1, 3). An increased intensity of conditioning represents opportunities to improve outcomes in these high-risk patients (2, 4). Studies have shown that relapse rates can be reduced by an intensified myeloablative conditioning regimen; however, the consequent high non-relapse mortality (NRM) counteracts any improvement in relapse (5, 6). Hence, exploring an effective conditioning regimen that does not significantly increase toxicity or NRM remains an important goal (7, 8).

Ruxolitinib is a JAK1/JAK2 inhibitor that inhibits tumor cell proliferation by suppressing JAK/STAT signal transduction (9, 10). A phase II study of ruxolitinib in patients with relapsed/refractory leukemia revealed that, after a median of two therapy cycles (ranging from 1 to 18), three patients achieved complete remission (CR), indicating its antileukemic efficacy and favorable tolerability when administered as monotherapy (9). Decitabine (5-aza-2-deoxycytidine) is a demethylating agent that inhibits DNA methyltransferases (11) and plays a crucial role in the treatment of AML and MDS (12). In particular, treatment with decitabine leads to a 13.9% reduction in relapse rates when used in combination with a busulfan-cyclophosphamide (Bu/Cy) conditioning regimen for patients with AML or MDS-IB (13). A recent HSCT trial in which decitabine-modified Bu/Cy conditioning was used in patients with relapsed/refractory AML reported that the cumulative incidence of relapse was 20% at two years (4). Ruxolitinib enhances the demethylating effect of decitabine on leukemia stem cells (LSCs) by inhibiting the JAK-STAT signaling pathway (14). We hypothesized that the combination of ruxolitinib and decitabine with mBu/Cy conditioning (Rux-Dec-mBu/Cy) would not increase toxicity and might lead to a reduction in relapse in AML/MDS patients.

We recently published the results of adding ruxolitinib and decitabine to a mBu/Cy regimen for allo-SCT in patients with high-risk AML for relapse prevention, which was well tolerated. The most common nonhematologic adverse event (AE) above grade 2 in severity was oropharyngeal mucositis (*n*=4, 10.8%), without increased NRM or impact engraftment. The overall survival (OS) and disease-free survival (DFS) rates at 1 year were 70.3% and 62.2%, respectively (3). Our previous research revealed that the Rux-dec-mBu/Cy regimen provided significant benefits for patients in their first complete remission (CR1). Therefore, this study included more CR1 patients than did previous studies, while also including some high-risk MDS patients. We further compared the safety and effectiveness of the intensified conditioning regimen with ruxolitinib and decitabine against the historical control regimen of mBu/Cy to explore the benefits of the new regimen. This expanded cohort analysis incorporates historical control cohorts, introducing a key methodological improvement, enabling a rigorous assessment of the efficacy of the combination of ruxolitinib and decitabine in the mBu/Cy conditioning regimen (Rux-Dec-mBu/Cy) for relapse prevention in high-risk AML/MDS patients, and providing comparative insights that were previously unavailable in prior studies.

Here, we present the safety, tolerability, and efficacy of a prospective, phase II trial testing the use of ruxolitinib and decitabine as part of the mBu/Cy conditioning regimen for patients with high-risk AML/MDS patients at the time of transplantation.

## Materials and methods

### Patient eligibility

Between May 2020 and July 2023, 58 high-risk AML and MDS patients were enrolled in this prospective phase II study. The study was approved by the Ethics Committee of the Chinese PLA General Hospital in accordance with the principles of the Declaration of Helsinki (ClinicalTrials.gov number NCT04582604). Written informed consent was obtained from all patients before enrollment.

The high-risk features of AML included CR1 accompanied by adverse genetic abnormalities (15), a refractory/relapsed status, or positive measurable residual disease (MRD) prior to transplantation. In Phase II, the protocol was amended (Version 1.1) to include intermediate/high-risk MDS patients, enabling efficacy evaluation of the intervention in this extended cohort. Additionally, the study included patients with high-risk MDS-IB (classified as high or very high by the Molecular International Prognostic Scoring System [IPSS-M]) (16).

**Figure 1:**
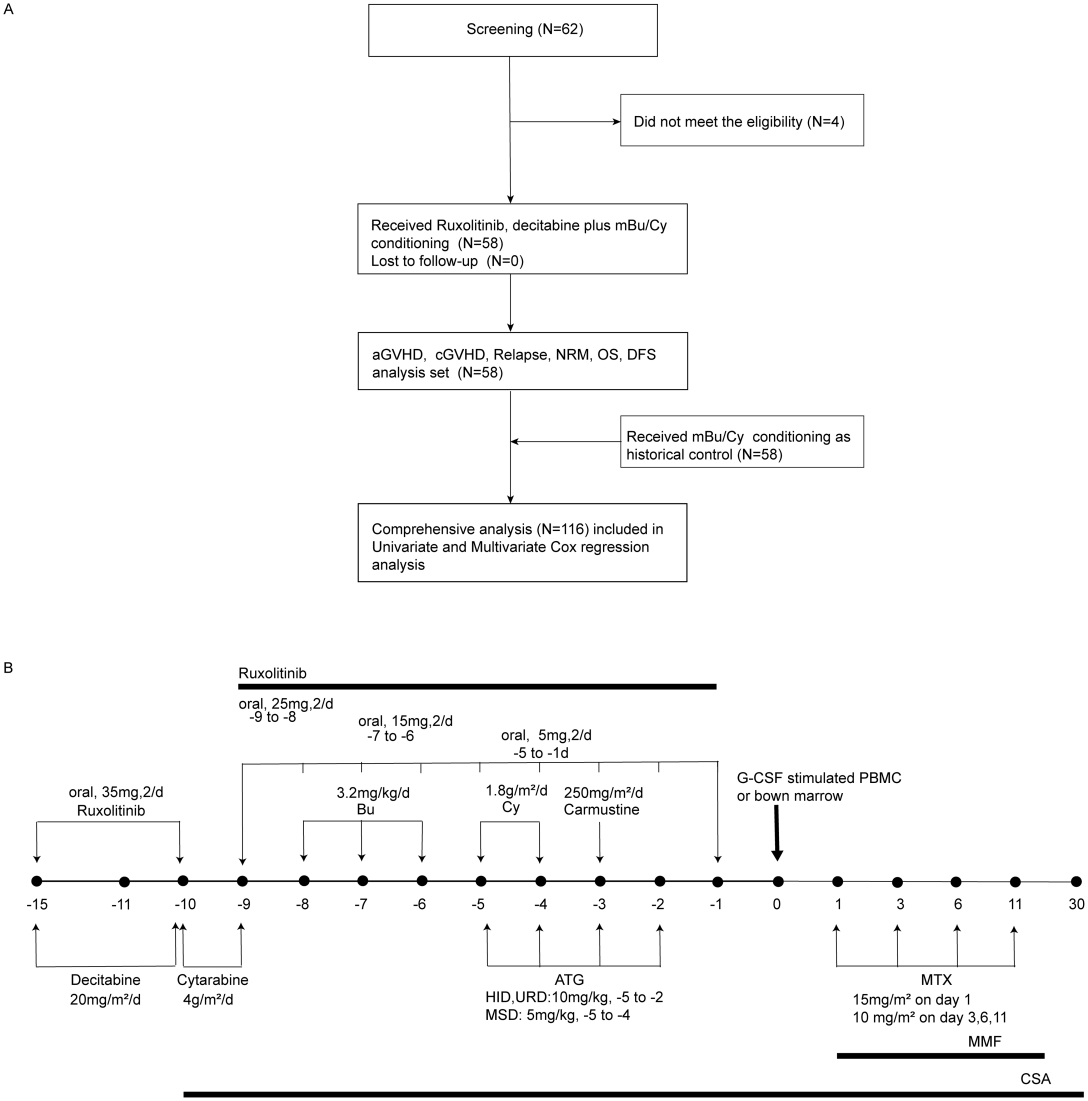
Flow chart of the trial and the administration of the conditioning regimen. **(A)** Flow chart of the trial. mBu/Cy, modified busulfan/cyclophosphamide; aGVHD, acute graft-versus-host disease; cGVHD, chronic graft-versus-host disease; NRM, non-relapse mortality; OS, overall survival; DFS, disease-free survival. **(B)** Ruxolitinib and decitabine combined with modified busulfan/cyclophosphamide (Rux-Dec-mBu/Cy): ruxolitinib, 35 mg twice daily, day −15 to -10, then tapering and discontinued on day −1; cytarabine, 4 g/m2/day, days −10 to -9, in unrelated donor or haploidentical hematopoietic stem cell transplantation, day −9, in matched sibling donor hematopoietic stem cell transplantation; busulfan, 3.2 mg/kg/day, day –8, cyclophosphamide, 1.8 g/m^2^/day, day –5, and carmustine, 250 mg/m^2^/day, day –3.

The exclusion criteria were as follows: patients with AML with t (15;17); patients with mental disorders or other states that rendered them unable to comply with the protocol; and pregnant females (**Figure 1A**).

A historical cohort comprising 58 patients (with the inclusion and exclusion criteria remaining the same as those in this trial) who received mBu/Cy consecutively from August 2018 to January 2022 was selected for reference to compare transplantation outcomes. The transplantation protocols in the historical cohort were the same as those in the phase II trial, including donor selection, graft-versus-host disease (GVHD) prophylaxis, and support therapy.

### Donor selection, conditioning regimen, and GVHD prophylaxis

HLA-matched sibling donors were the first option, followed by HLA-matched unrelated donors. If both donor types were unavailable, patients received a transplant from an HLA-haploidentical donor.

The conditioning regimen used was ruxolitinib (35 mg, orally, two times daily, day -15 to -10, then reduced by 10 mg every 2 days [day -9 to -6], 5 mg continued to day -1) (3, 17), decitabine (20 mg/m^2^)/day, day –15 ~ day –10), cytarabine (4 g/m^2^/day, day –10 ~ day –9 in HSCT with an unrelated matched donor [URD-HSCT], or HSCT with a haploidentical donor [HID-SCT], day –9 in HSCT with an HLA-matched sibling donor [MSD-HSCT]), busulfan (3.2 mg/kg/day, day –8 ~ day –6), cyclophosphamide (1.8 g/m^2^/day, day –5 ~ day –4), and carmustine (250 mg/m^2^/day, day –3) (18) (**Figure 1B**).

Cyclosporin A (2 mg/kg every 12 h via intravenous infusion, starting at day -10, tapered from 3 months post-transplant with discontinuation between 6–12 months, targeting trough concentrations of 150–250 μg/L), mycophenolate mofetil (30 mg/kg/day orally, initiated on day +1 and discontinued upon neutrophil engraftment), and short-term methotrexate (15 mg/m² on day +1 followed by 10 mg/m² on days +3, +6, and +11 via intravenous infusion) were used for GVHD prophylaxis (19). ATG (thymoglobulin, rabbit; Sanofi, Paris, France; 10 mg/kg, day –5~ day –2) was used for HID-HSCT and URD-HSCT. ATG, 5 mg/kg, day –5 ~ day –4, was used for MSD-HSCT. Supportive care was provided as described previously (20).

### Infection prophylaxis

Bacterial prophylaxis typically uses levofloxacin (500 mg/day during neutropenia). For antifungal prophylaxis, voriconazole (200 mg twice daily) or caspofungin (50 mg/day) is initiated pretransplant and maintained posttransplant, particularly during immunosuppressive therapy. Viral prophylaxis includes the use of ganciclovir (250 mg twice daily) to cover herpes simplex virus and cytomegalovirus (CMV), with CMV management prioritizes pretransplant PCR monitoring over universal prophylaxis. *Pneumocystis jirovecii* prophylaxis uses trimethoprim-sulfamethoxazole (TMP-SMX; 960 mg twice daily), starting 10 days before conditioning and continuing until immunosuppression cessation (≥6 months posttransplant).

### Study endpoints and definitions

The primary endpoint was the 2-year cumulative incidence of relapse. The secondary endpoints were the incidence rates of engraftment (+30 days), acute GVHD (+100 days), chronic GVHD (2-year), 2-year NRM, 2-year disease-free survival (DFS), 2-year graft-versus-host disease-free, relapse-free survival (GRFS), and 2-year overall survival (OS).

Relapse was defined as the reappearance of blasts in the blood in at least 2 peripheral blood samples at least 1 week apart, an increase in blasts to ≥5%, or the development of extramedullary disease after prior achievement of complete remission (CR). Neutrophil engraftment was defined as the first of 3 consecutive days with an absolute neutrophil count ≥ 0.5 × 10^9^/L. Platelet engraftment was defined as the first of 3 consecutive days with an absolute platelet count ≥ 20 × 10^9^/L without platelet transfusion for 7 days. The grades of acute GVHD were determined with the Mount Sinai Acute GVHD International Consortium (MAGIC) consensus (21), while chronic GVHD was diagnosed according to the American National Institute of Health (NIH) criteria (22). The definitions of NRM, DFS, and OS were the same as those outlined in previous studies (20).

### Adverse events and MRD management

Adverse events, including serious adverse events, were evaluated from the start of conditioning (day -15) to day +14. All adverse events were assessed via the National Cancer Institute Common Terminology Criteria for Adverse Events (CTCAE, version 5.0).

A multiparameter flow cytometry-measurable residual disease (MFC-MRD) assessment of the screening (pretransplant) and posttransplant MRD surveillance samples was conducted. We combined the leukemia-associated immunophenotype (LAIP); this approach differed from the normal strategy (DfN) for MRD detection, which incorporates core MRD markers to assess all samples (7).

### Post-transplant disease monitoring

Morphologic evaluation was conducted on bone marrow aspirates per standard cytomorphologic criteria. Multiparameter flow cytometry was employed to assess measurable residual disease (MRD), defining positivity as ≥0.1% CD45+ cells exhibiting leukemic immunophenotypes. Molecular profiling incorporated chimerism through short tandem repeat polymerase chain reaction (STR-PCR) using bone marrow (23). Serial assessments were conducted monthly during the first 3 post-transplant months, followed by evaluations at 6, 9, 12, 18, 24, 36, 48, and 60 months. Prophylactic donor lymphocyte infusion (DLI) is administered to patients with NR prior to transplantation. For matched sibling donors (MSDs), it is administered as early as +30 days; for haploidentical/unrelated donors, it is administered as early as +45 days. Donor lymphocytes are mobilized using G-CSF, contingent upon the absence of active GVHD. For patients with measurable residual disease (MRD)-positive status after transplantation, preemptive intervention with azacitidine followed by DLI is administered (3, 24). In patients with hematologic relapse after transplantation, G-CSF-mobilized lymphocytes were counted as CD3+ cells at 1 × 10^7^/kg in MSDs and 1–5 × 10^6^/kg in haploidentical/unrelated donors. The median dose of mononuclear cells (MNCs) for each infusion was 1.0 ×10^8^/kg, with a median CD3+ cell count of 1.0 ×10^7^/kg (24). Patients could receive repeated DLIs every 3–6 months depending on MRD and GVHD status after each infusion. Patients receiving DLIs from HIDs received CSA for 8 weeks after each infusion to prevent GVHD. Subjects receiving DLIs from matched sibling donors (MSDs) received CSA or methotrexate (MTX) for 4 weeks after each infusion to prevent GVHD (24).

### Sample size and statistical analysis

The sample size was calculated on the basis of the primary endpoint, the 2-year cumulative incidence of relapse, which was approximately 45% in patients with high-risk AML or MDS receiving allogeneic HSCT with Bu/Cy. To identify a 20% absolute decrease in the 2-year cumulative incidence of relapse with ruxolitinib, decitabine and mBu/Cy conditioning, a minimum of 58 patients (including 7% lost to follow-up) were required to provide the study with a significance level of 0.05 and a power of 80%. The sample size calculation was performed via PASS software (version 15.0). The sample size was recalculated during trial adaptation using updated relapse rates derived from interim survival analyses, per predefined protocol amendment thresholds (Protocol Version 1.1). The control group (n=58) included patients treated between August 2018 and January 2022 who received the mBu/Cy conditioning regimen. The study group (n=58) received the Rux-Dec-mBu/Cy conditioning regimen between May 2020 and July 2023.

The Mann–Whitney U test, X^2^ test, and Fisher’s exact probability test were used to compare the baseline characteristics between the Rux-Dec-mBu/Cy and historical control groups. The maximum grade for each type of AE was recorded for each patient. The cumulative incidence rates of NRM and relapse were estimated in the competing risk framework, with each being treated as a competing event. The cumulative incidence of acute and chronic GVHD was also estimated in the competing risk framework, with relapse or death without developing GVHD as a competing event. The Gray test was used for group comparisons of cumulative incidences. OS and DFS were estimated via the Kaplan–Meier method, and the log-rank test was used for group comparisons. Cox proportional hazard models were used to analyze relapse, NRM, OS and DFS. All *p* values were two-sided at the significance level of 0.05 unless otherwise stated. All analyses were performed via SPSS 22.0, EZR, and R version 4.2.3.

## Results

### Patient characteristics

Fifty-eight patients with high-risk leukemia or MDS were placed on the Rux-Dec-mBu/Cy conditioning regimen. No patients deviated from the protocol. Among the 58 patients treated (**Table 1**), the majority (n=44, 75.9%) were male, with a median age of 45 years (range: 15–67 years). Fifty-six patients had AML, 52 had primary AML, and the other 4 had secondary AML (3 with prior MDS and 1 with a prior history of breast cancer). Two additional patients were diagnosed with high-risk MDS-RAEB-2 based on the IPSS-M scoring system, and their bone marrow morphology assessments revealed 10.25% and 8.8% blasts, respectively. Next-generation sequencing (NGS) detected abnormalities in the TP53 gene (54.3%) and the DNMT3A gene (26.7%) in these 2 MDS patients.

**Table 1:** Patient and transplant characteristics.

Characteristic	Rux-Dec-mBu/Cy (n=58)	Historical control group (n=58)	*p*
**Patient’s age, years, median (range)**	45 (15-67)	34.5 (10-63)	0.31
Age ≥ 50, years, median (range)	21 (36.2%)	12 (20.7%)	0.06
**Male, %**	44 (75.9%)	38 (65.5%)	0.22
**Median WBC at diagnosis, ×10^9^/L (range)**	9.5 (1.0-336.6)	9.1 (0-295.0)	0.39
White blood cells≥100×10^9^/L, no (%)	8 (13.8%)	6 (10.3%)	0.57
Diagnosis			
Acute myeloid leukemia	56 (96.6%)	55(94.8%)	1.00
Myelodysplastic syndrome-IB	2 (3.4%)	3 (5.2%)	
**ELN, Risk stratification by genetics in AML**			0.51
Favorable	5 (8.9%)	7 (12.8%)	
Intermediate	19 (33.9%)	24 (43.6%)	
Adverse	32 (57.2%)	24 (43.6%)	
**MDS-IB**			1.00
High and very high	2 (100%)	3 (100%)	
**Disease status at transplantation, AML, no (%)**			0.06
Not treatment	2 (3.4%)	3 (5.2%)	
NR	14 (24.1%)	7 (12.0%)	
First complete remission (CR1)	31 (53.5%)	44 (75.8%)	
≥CR2	11 (19.0%)	4 (7.0%)	
Prior lines of therapy			
CR1			
Cycle to achieve CR	1 (1-4)	1 (1-4)	1.00
Consolidation cycle	1 (0-5)	2 (0-9)	0.07
≥CR2			
Cycle to achieve CR	1 (1-6)	2 (1-6)	0.96
Consolidation cycle	6 (3-9)	4 (1-6)	0.32
MRD before HSCT			
**First complete remission (CR1)**	31 (53.5%)	44 (75.8%)	0.74
MRD positive	15 (48.4%)	23 (52.3%)	
MRD negative	16 (51.6%)	21 (47.7%)	
**≥CR2**	11 (19.0%)	4 (7.0%)	1.00
MRD positive	6 (54.5%)	2 (50.0%)	
MRD negative	5 (45.5%)	2 (50.0%)	
**HCT-CI**			0.21
0	31 (53.4%)	39 (67.2%)	
1-2	23 (39.7%)	14 (24.2%)	
≥3	4 (6.9%)	5 (8.6%)	
**Donor’s age, years, median (range)**	34 (10-59)	39 (9-60)	0.34
**Male donor, %**	40 (69.0%)	41 (70.7%)	0.84
**Source of donors, no (%)**			0.58
Matched sibling donors	11 (19.0%)	14 (24.1%)	
Haploidentical donors	44 (75.8%)	43 (74.2%)	
Unrelated donors	3 (5.2%)	1 (1.7%)	
**ABO match, no (%)**			0.83
Match	30 (51.7%)	29 (50.0%)	
Major mismatch	10 (17.2%)	12 (20.7%)	
Minor mismatch	14 (24.1%)	11 (19.0%)	
Bidirectional mismatch	4 (7.0%)	6 (10.3%)	
**Graft source**			0.75
Peripheral blood	52 (89.7%)	53 (91.4%)	
Peripheral blood and bone marrow	6 (10.3%)	5 (8.6%)	
Graft			
MNCs, × 10^8^/kg	9.8 (4.4-23.4)	11.36 (5.2-28.0)	0.26
CD34+ cells, × 10^6/^kg	5.0 (0.8-21.6)	11.6 (2.1-29.0)	0.10
Treatment period	May 2020 - Jul 2023	Aug 2018 - Jan 2022	
Median follow-up time	967 (464-1597) d	1793 (1016-2157) d	

Rux-Dec-mBu/Cy, ruxolitinib and decitabine and the Bu/Cy conditioning regimen; CR, complete remission; CR1, complete remission after the first induction chemotherapy; CR2, complete remission at the second or later attempts; NR, nonremission; MRD, measurable residual disease; AML, acute myeloid leukemia; WBC, white blood cell; BM, bone marrow; PBSC, peripheral blood stem cell; MNC, mononuclear cell.; HCT-CI, HSCT comorbidity index

Bold values indicates the main categories of patient and transplant characteristics (such as patient age, percentage of males, median white blood cell count, etc.). Some main categories are followed by subcategories (such as the diagnostic classification includes acute myeloid leukemia and myelodysplastic syndrome-EB).

### Engraftment, regimen-related toxicity and graft-versus-host disease

Neutrophil engraftment was achieved in all patients, with sustained 100% donor chimerism confirmed by day +28 post-transplant. The median time for neutrophil recovery was 13 (9–21) days, whereas that for platelet recovery was 14 (7–65) days. The 30-day incidence rates of neutrophil and platelet engraftment were 100% and 94.8%, respectively. Platelet engraftment was not achieved in three patients by day +30. Two of these 3 patients achieved platelet engraftment on days +63 and +180, and 1 died on day +36 from acute GVHD grade IV without platelet engraftment.

The grade III-IV nonhematologic toxicities included oropharyngeal mucositis (n=5, 8.6%), diarrhea (n=3, 5.2%), nausea (n=1, 1.7%), rash (n=1, 1.7%), elevated aspartate aminotransferase (n=1, 1.7%), and elevated alanine aminotransferase (n=1, 1.7%) (**Table 2**). These adverse events resolved following symptomatic treatment. No deaths resulted from lethal organ toxicity as a result of Rux-Dec-mBu/Cy therapy in this study. The incidence of infection within 1 year after transplantation was 55.2% (n=32), with lung infections being the most common at 24.2% (n=14), followed by urinary tract infections at 10.3% (n=6) (**Supplementary Table 1**). Within 6 months post-transplantation, the cumulative incidence of cytomegalovirus (CMV) reactivation was 34.5% (95% CI 22.5-46.8%) (**Supplementary Figure 1A**), and the cumulative incidence of Epstein–Barr virus (EBV) reactivation was 62.1% (95% CI 48.1-73.3%) (**Supplementary Figure 1B**).

**Table 2:** Adverse events according to CTCAE version 5.0.

AE	Rux-Dec-mBu/Cy (n=58)		Historical control group (n=58)	
	Grades 1-2, no (%)	Grades ≥3, no (%)	Grades 1-2, no (%)	Grades ≥3, no (%)
Diarrhea	29 (50.0%)	3 (5.2%)	31 (53.4%)	6 (10.3%)
Nausea	28 (48.3%)	1 (1.7%)	36 (62.1%)	3 (5.2%)
Decreased appetite	30 (51.7%)	0 (0%)	29 (50.0%)	2 (3.4%)
Vomiting	16 (27.6%)	0 (0%)	20 (34.5%)	3 (5.2%)
Cough	3 (5.2%)	0 (0%)	4 (6.9%)	0 (0%)
Pyrexia	43 (74.1%)	0 (0%)	31 (53.4%)	1 (1.7%)
Headache	3 (5.2%)	0 (0%)	4 (6.9%)	0 (0%)
Abdominal pain	4 (6.9%)	0 (0%)	9 (15.5%)	0 (0%)
Fatigue	15 (25.9%)	0 (0%)	17 (29.3%)	2 (3.4%)
Constipation	4 (6.9%)	0 (0%)	4 (6.9%)	0 (0%)
Edema	2 (3.4%)	0 (0%)	1 (1.7%)	0 (0%)
Rash	12 (20.7%)	1 (1.7%)	12 (20.7%)	1 (1.7%)
Oropharyngeal mucositis	10 (17.2%)	5 (8.6%)	14 (24.2%)	7 (12.1%)
Increased aspartate aminotransferase	13 (22.4%)	1 (1.7%)	1 (1.7%)	0 (0%)
Increased alanine aminotransferase	17 (29.3%)	1 (1.7%)	6 (10.3%)	1 (1.7%)
Hypoalbuminemia	30 (51.7%)	0 (0%)	24(41.4%)	0 (0%)
Hypokalemia	37 (63.8%)	0 (0%)	33 (56.9%)	0 (0%)
Hypophosphatemia	12 (20.7%)	0 (0%)	14 (24.2%)	0 (0%)
Hypocalcemia	14 (24.2%)	0 (0%)	14 (24.2%)	0 (0%)

CTCAE, Common Terminology Criteria for Adverse Events. AE, adverse event.

**Figure 2:**
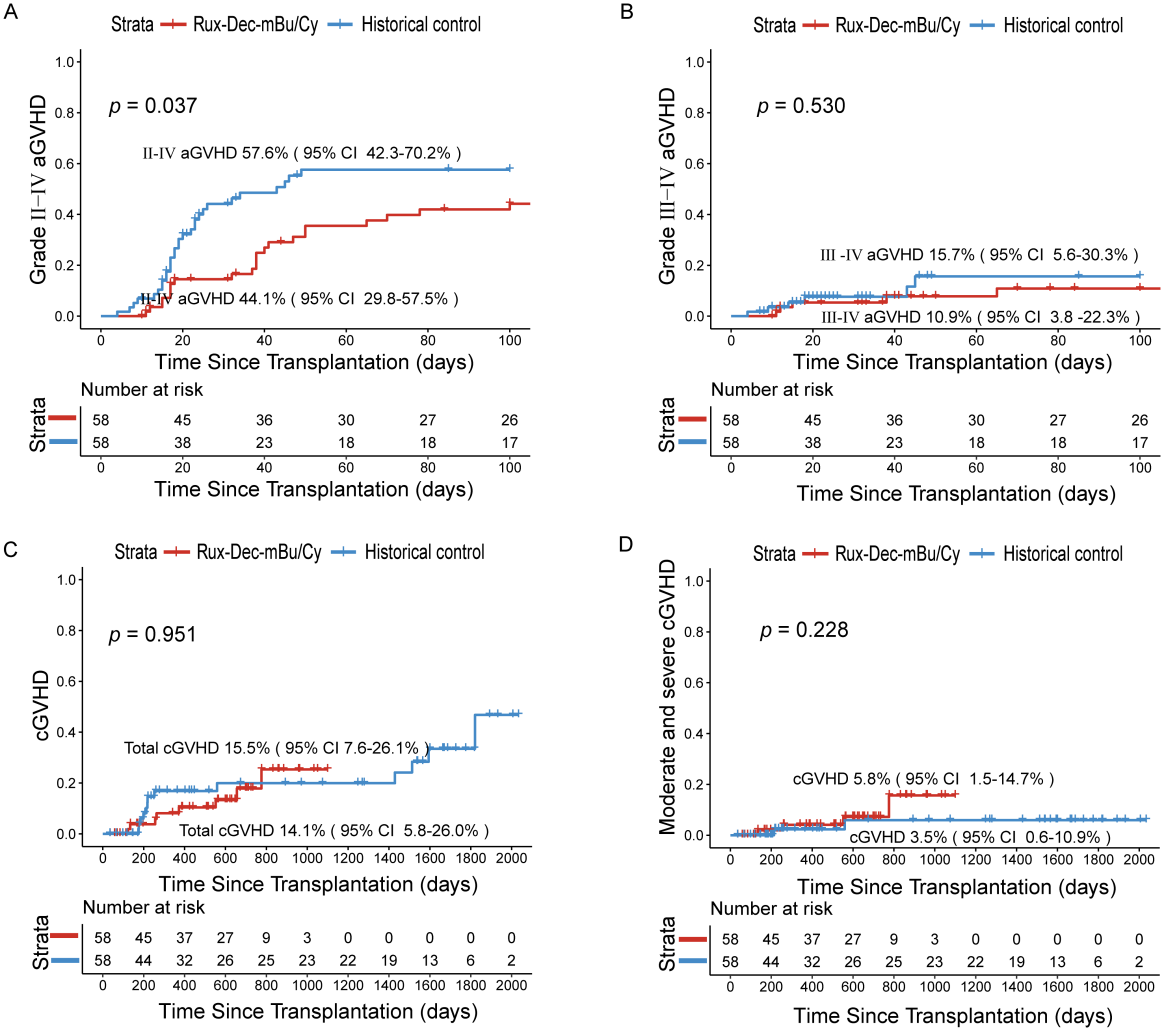
Cumulative incidence of aGVHD and cGVHD after treatment with ruxolitinib combined with decitabine plus the mBu/Cy conditioning regimen. **(A)** Cumulative incidence of II-IV aGVHD between Rux-Dec-mBu/Cy and the historical control at 100 days (p=0.037). **(B)** Cumulative incidence of III-IV aGVHD between Rux-Dec-mBu/Cy and the historical control at 100 days (p=0.530). **(C)** The 2-year cumulative incidence of total cGVHD between Rux-Dec-mBu/Cy and the historical control (p=0.951). **(D)** The 2-year cumulative incidence of moderate and severe cGVHD between Rux-Dec-mBu/Cy and the historical control (p=0.228). aGVHD, acute graft-versus-host disease; cGVHD, chronic graft-versus-host disease; Rux-Dec-mBu/Cy, ruxolitinib and decitabine plus mBu/Cy conditioning regimen; 95% CI, 95% confidence interval.

The cumulative incidence of grade II–IV aGVHD was 44.1% (95% CI: 29.8–57.5%) (**Figure 2A**), and that of grade III–IV acute GVHD was 10.9% (95% CI: 3.8–22.3%) (**Figure 2B**). The cumulative incidence of total chronic GVHD at 2 years was 14.1% (95% CI: 5.8–26.0%) (**Figure 2C**), whereas the cumulative incidence of moderate and severe chronic GVHD reached 5.8% (95% CI: 1.5–14.7%) (**Figure 2D**).

### Relapse and non-relapse mortality

**Figure 3:**
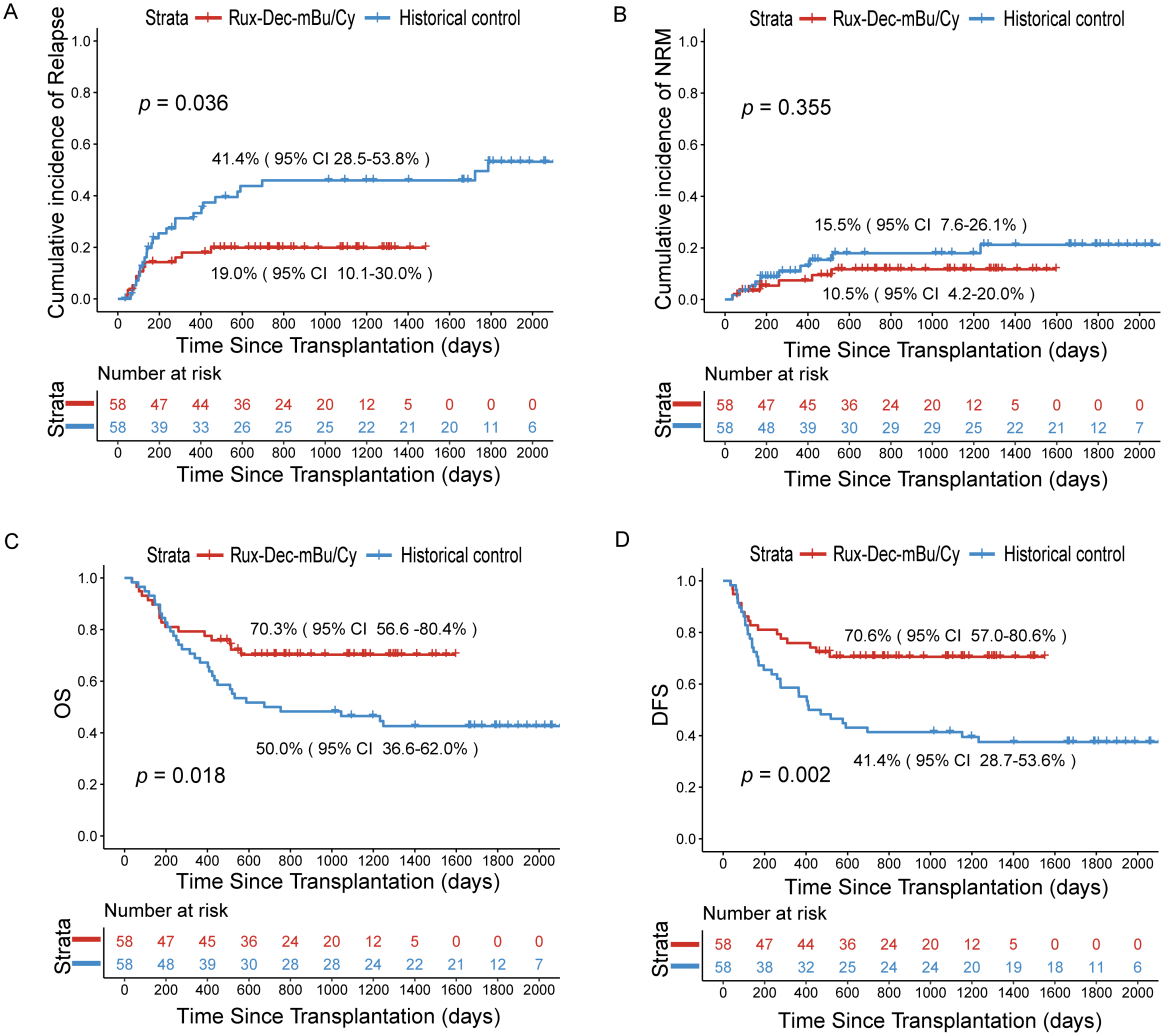
Cumulative incidence of relapse, NRM, OS, and DFS after the ruxolitinib and decitabine plus mBu/Cy conditioning regimen. **(A)** Comparison of the relapse rates of Rux-Dec-mBu/Cy and the historical control at 2 years (p=0.036). **(B)** Comparison of the NRM rates of Rux-Dec-mBu/Cy and the historical control at 2 years (p=0.355). **(C)** Comparison of the OS rates of Rux-Dec-mBu/Cy and the historical control at 2 years (p=0.018). **(D)** Comparison of the DFS rates of Rux-Dec-mBu/Cy and the historical control at 2 years (p=0.002). Rux-Dec-mBu/Cy, ruxolitinib and decitabine plus the mBu/Cy conditioning regimen; NRM, non-relapse mortality; OS, overall survival; DFS, disease-free survival; 95% CI, 95% confidence interval.

Relapse occurred in 12 patients. The 2-year cumulative incidence of relapse was 19.0% (95% CI: 10.1–30.0%) (**Figure 3A**). The 2-year cumulative incidence of relapse in the CR1 group was 3.2% (95% CI: 0.2–14.4%), and that in the ≥CR2 group was 37.0% (95% CI: 19.2–55.0%) (*p*<0.001) (**Supplementary Figure 2A**). The 2-year cumulative incidence in the MRD-negative group prior to conditioning was 0, and that in the MRD-positive group was 19.0% (95% CI: 5.7–38.3%) (*p* = 0.038) (**Supplementary Figure 2B**).

Forty-one patients in the Rux-Dec-mBu/Cy group were alive by the time of analysis, which was December 2024. The 2-year cumulative incidence of NRM was 10.5% (95% CI, 4.2–20.0%) (**Figure 3B**). The causes of NRM included severe infections in 4 patients. Two additional patients died from aGVHD: one of the patients had grade IV aGVHD (stage 4 lower gastrointestinal involvement) and the other had grade IV aGVHD (stage 4 lower gastrointestinal involvement with stage 1 upper gastrointestinal involvement).

### Survival

After a median follow-up period of 967 days (range, 464–1597 days), 17 (29.3%) patients died. The 2-year overall survival estimate was 70.3% (95% CI: 56.6%-80.4%) (**Figure 3C**). The 2-year probability of DFS was 70.6% (95% CI: 57.0%-80.6%) (**Figure 3D**). The 2-year probability of GRFS was 65.2% (95% CI: 51.4%-76.0%). The 2-year overall survival probability was 86.7% (95% CI: 68.3-94.8%) for patients with CR1, whereas it was 51.4% (95% CI: 31.3-68.2%) for patients with ≥CR2 (*p*=0.003) (**Supplementary Figure 2C**). In addition, the 2-year overall survival probability was 90.5% (95% CI: 67.0-97.5%) in the MRD-negative group prior to conditioning and 71.1% (95% CI: 46.6-85.9%) in the MRD-positive group (*p*=0.120) (**Supplementary Figure 2D**).

### Historical control cohort

Although this study was not randomized, we compared the outcomes of patients who received conventional conditioning (mBu/Cy) before transplantation, which served as a “historical control (N = 58)”. Compared with that in the historical control group, the 2-year cumulative incidence of relapse was significantly lower (Rux-Dec-mBu/Cy group: 19.0% [95% CI: 10.1%–30.0%]; historical control: 41.4% [95% CI: 28.5%–53.8%], *p*=0.036; **Figure 3A**). Subgroup analysis found that the Rux-Dec-mBu/Cy group had a lower 2-year cumulative incidence of relapse compared to the historical group in CR1 (3.2% (95% CI: 0.2%–14.4%) vs 36.4% (95% CI: 22.4%–50.5%), *p* < 0.001) (**Supplementary Figure 3A**). There were no significant differences in CR2 (**Supplementary Figure 3B**). The cumulative incidence of grade II-IV aGVHD was significantly lower (Rux-Dec-mBu/Cy group: 44.1% [95% CI: 29.8–57.5%]; historical control: 57.6% [95% CI: 42.3–70.2%], *p*=0.037; **Figure 2A**). No statistically significant differences in grade III-IV acute GVHD (**Figure 2B**), chronic GVHD (**Figure 2C**), or moderate or severe chronic GVHD (**Figure 2D**) between the Rux-Dec-mBu/Cy group and the historical control group were noted. Moreover, no statistically significant difference was observed in the cumulative incidence of NRM at 2 years (**Figure 3B**). Overall, the comparison of the 2-year OS (Rux-Dec-mBu/Cy group: 70.3% [95% CI: 56.6–80.4%]; historical control: 50.0% [95% CI: 36.6%–62.0%], *p* =0.018; **Figure 3C**), 2-year DFS (Rux-Dec-mBu/Cy group: 70.6% [95% CI: 57.0%–80.6%]; historical control: 41.4% [95% CI: 28.7%–53.6%], *p* =0.002; **Figure 3D**), and 2-year GRFS (Rux-Dec-mBu/Cy group: 65.2% [95% CI: 51.4%–76.0%]; historical control: 31.0% [95% CI: 19.7%–43.0%], *p* < 0.001) between the two cohorts revealed a significant difference in survival.

Among haplo-HSCT recipients, the 2-year cumulative incidence of relapse was lower with Rux-Dec-mBu/Cy versus the control group (20.5% vs. 32.6%; *p*=0.287), though this difference did not reach statistical significance, potentially due to limited sample size. Conversely, Rux-Dec-mBu/Cy demonstrated a significantly reduced cumulative incidence of grade II-IV aGVHD compared to historical controls (42.0% vs. 65.1%; *p*=0.007) (**Supplementary Table 2**).

### Comprehensive analysis

**Figure 4:**
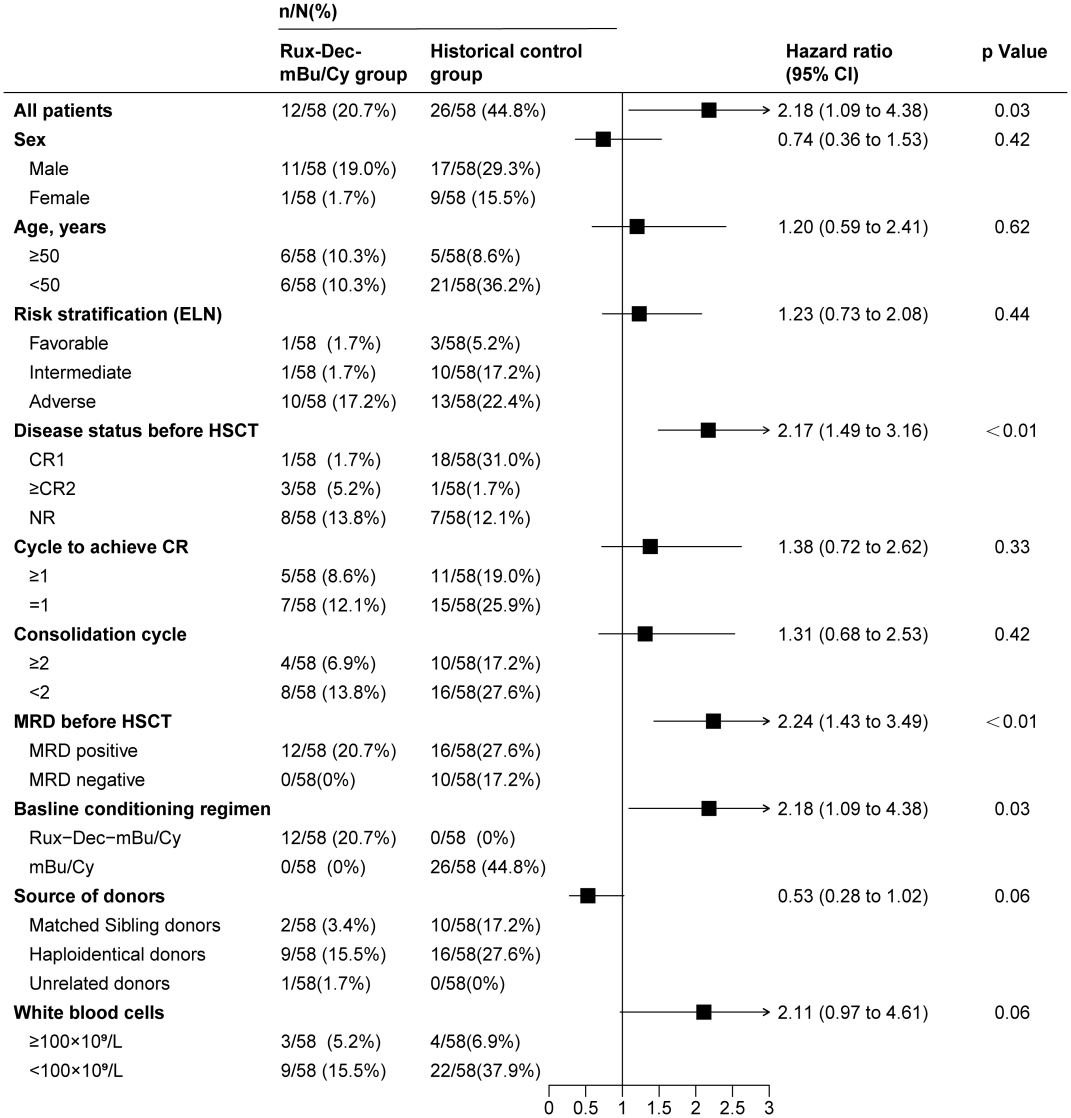
Univariate analysis of factors associated with relapse in high-risk AML patients after allo-HSCT. Rux-Dec-mBu/Cy, ruxolitinib and decitabine plus the mBu/Cy conditioning regimen; ELN, European Leukemia Net 2017 classification; HSCT, allogeneic hematopoietic stem cell transplantation; CR, complete remission; CR1, complete remission after the first induction chemotherapy; CR2, complete remission at the second or later attempts; NR, nonremission; MRD, measurable residual disease; 95% CI, 95% confidence interval.

A consistent pattern of a lower cumulative incidence of relapse in the Rux-Dec-mBu/Cy group than in the historical control group was noted across multiple subgroups (**Figure 4**). Multivariate analysis revealed that Rux-Dec conditioning (HR=3.22, 95% CI: 1.50–6.91; *p* < 0.01), complete remission after the first induction chemotherapy (CR1) before HSCT (HR=2.20, 95% CI: 1.15–4.22; *p* =0.02) and white blood cell count<100×10^9^/L (HR=3.18, 95% CI: 1.38–7.31; *p* < 0.01) were protective factors against the cumulative incidence of relapse (**Supplementary Table 3**). Multivariate analysis revealed that the Rux-Dec-mBu/Cy conditioning regimen was an independent protective factor influencing OS (HR=2.14, 95% CI: 1.14-4.02; *p*=0.02) and DFS (HR=2.68, 95% CI: 1.45-4.99; *p* < 0.01) (**Supplementary Tables 4**, **5**).

## Discussion

This study revealed that Rux-Dec-mBu/Cy results in good outcomes which compare favorably with historical controls using the same protocol without ruxolitinib and decitabine in patients with high-risk acute myeloid leukemia or MDS who are undergoing allogeneic HSCT. Non-relapse mortality was similar between the two groups; these results are probably attributable to better antileukemeic activity and less relapse rates. Tolerability benchmarks such as regimen-related toxicity and grade 3 or worse adverse events were similar between the two groups.

Novel conditioning regimens are gaining traction, including venetoclax-based combinations to enhance myeloid suppression (25), total marrow irradiation (TMI) for targeted dose escalation (26), and ^131^I-apamistamab followed by Flu-TBI to exploit antibody-mediated immune modulation (8, 27). Within the recent EBMT conditioning intensity framework (28), our Rux-Dec-mBu/Cy conditioning regimen aligns with the intermediate TCI category (score range: 2.5–3.5), positioned between low- (1–2) and high-intensity (4–6) classifications. This intermediate-intensity profile offers a clinically viable strategy for high-risk patients requiring balanced efficacy and tolerability.

Relapse is a leading cause of transplant failure, with patients being particularly at risk of relapse within the first year after transplantation, and the cumulative incidence of relapse is reported to be between 40% and 50% (29–31). Conditioning regimens are an essential factor affecting relapse after transplantation. For patients with high-risk AML or MDS, the cumulative incidence of relapse was approximately 50% in patients who received reduced-intensity conditioning and 30% in patients who received myeloablative conditioning (5, 32). In our study, Rux-Dec-mBu/Cy reduced cumulative incidence of relapse compared with that in our historical control group. These findings suggest that the combined regimen may offer a new option for patients with high-risk AML and MDS before allo-HSCT.

Ciurea et al. (33) analyzed 1349 AML patients who received allo-HSCT from the Center for International Blood and Marrow Transplant Research (CIBMTR) (*De novo* AML, 84%; CR, 66%; relapse, 34%; adverse-risk cytogenetic abnormalities, 22%) and reported that the 2-year cumulative incidence of relapse was 44% in haploidentical donors and 39% in unrelated donors. Tang et al. (4) reported that the addition of decitabine to mBu/Cy conditioning resulted in a 2-year cumulative incidence of relapse of approximately 20% (n=52) in high-risk AML patients. Similarly, the relapse rate for the mBu/Cy-only group was 45% (n=177). Recently, we presented a single-arm, prospective study of 37 high-risk AML patients who received ruxolitinib and decitabine combined with a modified Bu/Cy regimen, and our findings demonstrated the safety and initial efficacy of this novel treatment approach (3). The current study revealed that the 2-year cumulative incidence of relapse were 19.0% and 41.4% in the historical group (*p*=0.036). This improvement in relapse translated into a survival advantage, with a 2-year overall survival of 70.3%, which was superior to the 50.0% overall survival in the historical control and that reported in the literature (mBuCy group) (4, 33). The specific mechanism is worthy of further investigation.

After HSCT with standard preventive measures, aGVHD can occur in 50–70% of patients, posing a significant challenge to achieving favorable transplantation results (34). Ruxolitinib has been granted approval by the US Food and Drug Administration (FDA) as the exclusive treatment option for patients with aGVHD who do not respond to steroid therapy (35). Currently, there is no research exploring the effects of using ruxolitinib in pretransplant conditioning regimens. In the present study, we incorporated ruxolitinib into the mBu/Cy conditioning regimen, considering that ruxolitinib affects the differentiation, phenotype, and function of dendritic cells, leading to impaired T-cell activation (36). In this study, the cumulative incidence of grade II-IV aGVHD was 44.1% with the Rux-Dec-mBu/Cy regimen, which was lower than the historical control group rate of 57.6% (*p* =0.037). In a phase II study conducted by Eghtedar et al. (9), ruxolitinib, when given to patients with relapsed or refractory leukemias, including *de novo* acute myeloid leukemia (AML) and secondary AML, showed some antileukemic effects and was found to be well tolerated as a single-agent therapy. In our study, ruxolitinib was added to the mBu/Cy conditioning regimen, and it improved the aGVHD.

Our analysis highlights the important therapeutic rationale for integrating JAK/STAT inhibitors (ruxolitinib) with hypomethylating therapy (decitabine) into the mBu/Cy conditioning regimen. *In vivo* administration of hypomethylating agents mitigate GVHD without sacrificing graft-versus-leukemia (37). Yang et al. (38) suggests that ruxolitinib may help alleviate excessive inflammation by directing macrophage activity towards a more regulated pattern. The synergistic potential of this novel conditioning regimen lies in its dual targeting of inflammatory pathways (38) and epigenetic regulation (37)—both of which are key drivers of GVHD and relapse in allo-HSCT patients. Ruxolitinib may improve GVHD by reducing the release of inflammatory factors after transplantation. Our study shows that Rux-Dec-mBu/Cy reduces recurrence after transplantation while also improving GVHD. Notably, the preliminary efficacy demonstrated in the haploidentical transplant cohort is encouraging, suggesting that this regimen may enhance graft-versus-leukaemia effects without exacerbating regimen-related toxicity.

More importantly, the results presented here show the safety of using Rux-Dec-mBu/Cy allo-HSCT in patients with high-risk MDS and AML. Engraftment was not delayed after the delivery of ruxolitinib and decitabine, and the conditioning-related toxicities were mild and manageable. Several studies (39, 40), including our retrospective study (41), reported that mBu/Cy conditioning resulted in a 2-year cumulative incidence of NRM of approximately 20%. The 2-year cumulative incidence of NRM did not differ between the Rux-Dec-mBu/Cy regimen group and the historical control group (10.5% vs. 15.5%, *p*=0.355). Most deaths (n=4) were attributable to infections. Although our non-relapse mortality results were no statistically difference with those of the historical control group, the non-relapse mortality rate was lower than that previously reported. Thus, it would be interesting in the future to confirm these results in a larger cohort and to compare the Rux-Dec-mBu/Cy regimen prospectively to the results of other widely used mBu/Cy regimens in the particular setting of patients with MDS/AML to demonstrate its superiority.

The limitations of this study include inherent limitations of historical control groups, where heterogeneous patient selection and the introduction of evolving supportive therapies introduce confounding variables that may have exaggerated treatment progress. The non-randomized design and lack of comprehensive immune monitoring further emphasize the necessity of validation in future prospective trials. Our focus on early adverse events (up to Day +14) could miss later toxicities like prolonged cytopenias. Thus, studies with longer follow-up are needed to fully define long-term safety. To clearly establish the superiority over standard conditioning regimens and overcome the above limitations, our center has initiated a prospective, randomized controlled trial (RCT) comparing the novel Rux-Dec-mBu/Cy regimen with the traditional mBu/Cy regimen. Additionally, the impact of the Rux-Dec-mBu/Cy regimen on patients with a response of complete remission at the second or later attempts (≥CR2) was not as satisfactory as that observed in patients who achieved a CR1. Further research and functional studies on the antileukemic effects of ruxolitinib should be conducted to confirm the efficacy of Rux-Dec-mBu/Cy regimen in high-risk AML/MDS patients.

In conclusion, our study indicates that the Rux-Dec-mBu/Cy regimen, may be an option for patients with high-risk AML/MDS undergoing allo-HSCT. This approach is particularly beneficial for patients who have achieved CR1 prior to conditioning. The findings from our study could inform the planning of allogeneic HSCT strategies for individuals with high-risk AML/MDS.

## Data Availability

The authors declare that all data supporting the findings of this study are available in the article and **Supplementary Material** (https://www.frontiersin.org/articles/10.3389/fimmu.2025.1586512/full#supplementary-material). The raw data supporting the findings of this study can be requested from the corresponding author upon reasonable request.
