# Supplementary material for: Ruxolitinib and decitabine plus a busulfan–cyclophosphamide conditioning regimen for relapse prophylaxis in patients with high-risk acute myeloid leukemia or myelodysplastic syndromes
Source: Front Immunol. 2025 Aug 18;16:1586512. doi: 10.3389/fimmu.2025.1586512 (PMC12399409; doi:10.3389/fimmu.2025.1586512)

**Supplementary Table 1. Outcomes after allogeneic hematopoietic stem cell transplantation**

| **Variable** | **Rux-Dec-mBu/Cy (n=58)** | **Historical control group (n=58)** | *p* |
| --- | --- | --- | --- |
| **Engraftment** |  |  |  |
| Neutrophil engraftment | 58 (100.0%) | 58 (100.0%) | 1.00 |
| Platelet engraftment | 55 (94.8%) | 57 (98.3%) | 0.62 |
| **Infections, no(%)** | 32 (55.2%) | 31 (53.4%) | 0.37 |
| Lung infections, no(%) | 14 (24.2%) | 20 (34.5%) |  |
| Intestinal infections, no(%) | 5 (8.6%) | 4 (6.9%) |  |
| Urinary tract infection, no(%) | 6 (10.3%) | 1 (1.7%) |  |
| Skin and soft tissue infections, no(%) | 2 (3.4%) | 3 (5.2%) |  |
| Sepsis, no(%) | 5 (8.6%) | 3 (5.2%) |  |
| **Post-transplantation lymphoproliferative disorder, no(%)** | 8 (13.8%) | 4 (6.9%) | 0.36 |
| **Hemorrhagic cystitis, no(%)** | 19 (32.8%) | 18 (31.0%) | 0.96 |
| Ⅰ | 15 (25.9%) | 13 (22.4%) |  |
| II | 1 (1.7%) | 2 (3.4%) |  |
| Ⅲ | 3 (5.2%) | 3 (5.2%) |  |
| IV | 0 | 0 |  |
| **DLI** | 4 (6.9%) | 5 (8.6%) |  |

Rux-Dec-mBu/Cy, ruxolitinib and decitabine and mBu/Cy conditioning regimen; CR, complete remission; DLI, donor lymphocyte infusion.

**Supplementary Table 2. Transplant outcomes in patients receiving haploidentical allogeneic haematopoietic stem cell transplantation**

|  | 2-year Relapse | 2-year  NRM | 2-year  DFS | 2-year  OS | II–IV aGVHD | 2-year  cGVHD |
| --- | --- | --- | --- | --- | --- | --- |
| Rux-Dec-mBu/Cy (n=44) | 20.5% (10.0-33.5) | 6.8%  (1.7-16.9) | 72.7% (57.0-83.5) | 72.6% (56.8-83.4) | 42.0% (25.3-57.9) | 13.6% (4.5-27.8) |
| Historical control  (n=43) | 32.6% (19.1-46.8) | 16.3  (7.1-28.9) | 48.8% (33.3-62.6) | 55.8% (39.8%-69.1) | 65.1% (45.7-79.0) | 14.0% (5.6-26.1) |
| *p* | 0.287 | 0.146 | 0.029 | 0.087 | 0.007 | 0.881 |

Rux-Dec-mBu/Cy, ruxolitinib and decitabine and mBu/Cy conditioning regimen; NRM, non-relapse mortality; OS, overall survival; DFS, disease-free survival; aGVHD, acute graft-versus-host disease; cGVHD, chronic graft-versus-host disease

**Supplementary Table 3. Univariate and multivariate analysis of factors associated with outcomes of high-risk AML and MDS patients after allo-HSCT (Relapse).**

| **Variables** | **Univariate analysis** | | | **Multivariate analysis** | | |
| --- | --- | --- | --- | --- | --- | --- |
|  | **HR** | **95%CI** | ***p*** | **HR** | **95%CI** | ***p*** |
| **Patient age** | 1.20 | 0.59-2.41 | 0.62 |  |  |  |
| ≥50 |  |  |  |  |  |  |
| <50 |  |  |  |  |  |  |
| **Sex** | 0.74 | 0.36-1.53 | 0.42 |  |  |  |
| Male |  |  |  |  |  |  |
| Female |  |  |  |  |  |  |
| **Risk stratification (ELN)** | 1.23 | 0.73-2.08 | 0.44 |  |  |  |
| Favorable |  |  |  |  |  |  |
| Intermediate |  |  |  |  |  |  |
| Adverse |  |  |  |  |  |  |
| **Disease status before HSCT** | 2.17 | 1.49-3.16 | **＜0.01** | 2.20 | 1.15-4.22 | 0.02 |
| CR1 |  |  |  |  |  |  |
| ≥CR2 |  |  |  |  |  |  |
| NR |  |  |  |  |  |  |
| **Cycle to achieve CR** | 1.38 | 0.72-2.62 | 0.33 |  |  |  |
| ≥2 |  |  |  |  |  |  |
| ＜1 |  |  |  |  |  |  |
| **Consolidation cycle** | 1.31 | 0.68-2.53 | 0.42 |  |  |  |
| ≥2 |  |  |  |  |  |  |
| ＜2 |  |  |  |  |  |  |
| **MRD before HSCT** | 2.24 | 1.43-3.49 | **＜0.01** | 1.29 | 0.62-2.71 | 0.49 |
| MRD positive |  |  |  |  |  |  |
| MRD negative |  |  |  |  |  |  |
| **Basline conditioning regimen** | 2.18 | 1.09-4.38 | **0.03** | 3.22 | 1.50-6.91 | **＜0.01** |
| Rux-Dec-intensified |  |  |  |  |  |  |
| mBu/Cy |  |  |  |  |  |  |
| **Source of donors** | 0.53 | 0.28-1.02 | **0.06** | 0.63 | 0.32-1.23 | **0.17** |
| Matched Sibling donors |  |  |  |  |  |  |
| Haploidentical donors |  |  |  |  |  |  |
| Unrelated donors |  |  |  |  |  |  |
| **White blood cells** | 2.11 | 0.97-4.61 | **0.06** | 3.18 | 1.38-7.31 | **＜0.01** |
| ≥100×10^9^/L |  |  |  |  |  |  |
| ＜100×10^9^/L |  |  |  |  |  |  |

Rux-Dec-mBu/Cy, ruxolitinib and decitabine and mBu/Cy conditioning regimen; ELN, European Leukemia Net 2017 classification; HSCT, allogeneic hematopoietic stem cell transplantation, CR, complete remission; CR1, Achieved complete remission after first induction chemotherapy; CR2, complete remission at the second or later attempts; NR, Non-remission; MRD, Measurable residual disease; 95% CI, 95% confidence interval.

**Supplementary Table 4. Univariate and multivariate analysis of factors associated with outcomes of high-risk AML and MDS patients after allo-HSCT (OS).**

| **Variables** | **Univariate analysis** | | | **Multivariate analysis** | | |
| --- | --- | --- | --- | --- | --- | --- |
|  | **HR** | **95%CI** | ***p*** | **HR** | **95%CI** | ***p*** |
| **Patient age** | 1.43 | 0.80-2.56 | 0.23 |  |  |  |
| ≥50 |  |  |  |  |  |  |
| <50 |  |  |  |  |  |  |
| **Sex** | 0.83 | 0.45-1.55 | 0.56 |  |  |  |
| Male |  |  |  |  |  |  |
| Female |  |  |  |  |  |  |
| **Risk stratification (ELN)** | 0.92 | 0.58-1.45 | 0.71 |  |  |  |
| Favorable |  |  |  |  |  |  |
| Intermediate |  |  |  |  |  |  |
| Adverse |  |  |  |  |  |  |
| **Disease status before HSCT** | 1.73 | 1.23-2.42 | **＜0.01** | 1.42 | 0.83-2.43 | 0.21 |
| CR1 |  |  |  |  |  |  |
| ≥CR2 |  |  |  |  |  |  |
| NR |  |  |  |  |  |  |
| **Cycle to achieve CR** | 1.28 | 0.73-2.25 | 0.39 |  |  |  |
| ≥1 |  |  |  |  |  |  |
| ＜1 |  |  |  |  |  |  |
| **Consolidation cycle** | 1.24 | 0.69-2.20 | 0.47 |  |  |  |
| ≥1 |  |  |  |  |  |  |
| ＜1 |  |  |  |  |  |  |
| **MRD before HSCT** | 2.00 | 1.37-2.91 | **＜0.01** | 1.53 | 0.82-2.87 | 0.18 |
| MRD positive |  |  |  |  |  |  |
| MRD negative |  |  |  |  |  |  |
| **Basline conditioning regimen** | 2.00 | 1.11-3.60 | **0.02** | 2.14 | 1.14-4.02 | **0.02** |
| Rux-Dec-intensified |  |  |  |  |  |  |
| mBu/Cy |  |  |  |  |  |  |
| **Source of donors** | 0.74 | 0.42-1.31 | 0.30 |  |  |  |
| Matched Sibling donors |  |  |  |  |  |  |
| Haploidentical donors |  |  |  |  |  |  |
| Unrelated donors |  |  |  |  |  |  |
| **White blood cells** | 1.23 | 0.55-2.74 | 0.61 |  |  |  |
| ≥100×10^9^/L |  |  |  |  |  |  |
| ＜100×10^9^/L |  |  |  |  |  |  |

Rux-Dec-mBu/Cy, ruxolitinib and decitabine and mBu/Cy conditioning regimen; ELN, European Leukemia Net 2017 classification; HSCT, allogeneic hematopoietic stem cell transplantation, CR, complete remission; CR1, Achieved complete remission after first induction chemotherapy; CR2, complete remission at the second or later attempts; NR, Non-remission; MRD, Measurable residual disease; 95% CI, 95% confidence interval.

**Supplementary Table 5. Univariate and multivariate analysis of factors associated with outcomes of high-risk AML and MDS patients after allo-HSCT (DFS).**

| **Variables** | **Univariate analysis** | | | **Multivariate analysis** | | |
| --- | --- | --- | --- | --- | --- | --- |
|  | **HR** | **95%CI** | ***p*** | **HR** | **95%CI** | ***p*** |
| **Patient age** | 1.32 | 0.74-2.35 | 0.35 |  |  |  |
| ≥50 |  |  |  |  |  |  |
| <50 |  |  |  |  |  |  |
| **Sex** | 0.84 | 0.46-1.53 | 0.57 |  |  |  |
| Male |  |  |  |  |  |  |
| Female |  |  |  |  |  |  |
| **Risk stratification (ELN)** | 0.98 | 0.63-1.52 | 0.92 |  |  |  |
| Favorable |  |  |  |  |  |  |
| Intermediate |  |  |  |  |  |  |
| Adverse |  |  |  |  |  |  |
| **Disease status before HSCT** | 1.73 | 1.25-2.41 | **＜0.01** | 1.60 | 0.95-2.70 | 0.08 |
| CR1 |  |  |  |  |  |  |
| ≥CR2 |  |  |  |  |  |  |
| NR |  |  |  |  |  |  |
| **Cycle to achieve CR** | 1.29 | 0.75-2.23 | 0.36 |  |  |  |
| ≥1 |  |  |  |  |  |  |
| ＜1 |  |  |  |  |  |  |
| **Consolidation cycle** | 1.25 | 0.71-2.19 | 0.44 |  |  |  |
| ≥1 |  |  |  |  |  |  |
| ＜1 |  |  |  |  |  |  |
| **MRD before HSCT** | 1.89 | 1.31-2.73 | **＜0.01** | 1.34 | 0.74-2.44 | 0.33 |
| MRD positive |  |  |  |  |  |  |
| MRD negative |  |  |  |  |  |  |
| **Basline conditioning regimen** | 2.42 | 1.35-4.31 | **＜0.01** | 2.68 | 1.45-4.99 | **＜0.01** |
| Rux-Dec-intensified |  |  |  |  |  |  |
| mBu/Cy |  |  |  |  |  |  |
| **Source of donors** | 0.66 | 0.38-1.15 | 0.14 |  |  |  |
| Matched Sibling donors |  |  |  |  |  |  |
| Haploidentical donors |  |  |  |  |  |  |
| Unrelated donors |  |  |  |  |  |  |
| **White blood cells** | 1.57 | 0.74-3.33 | 0.24 |  |  |  |
| ≥100×10^9^/L |  |  |  |  |  |  |
| ＜100×10^9^/L |  |  |  |  |  |  |

Rux-Dec-mBu/Cy, ruxolitinib and decitabine and mBu/Cy conditioning regimen; ELN, European Leukemia Net 2017 classification; HSCT, allogeneic hematopoietic stem cell transplantation, CR, complete remission; CR1, Achieved complete remission after first induction chemotherapy; CR2, complete remission at the second or later attempts; NR, Non-remission; MRD, Measurable residual disease; 95% CI, 95% confidence interval.

**Supplementary Figure Legends**

**Supplementary Figure 1. The cumulative incidence of CMV and EBV at 6 months.** (A) Comparison of the cumulative incidence of CMV between Rux-Dec-mBu/Cy and historical control at 6 months (*p*=0.520), (B) Comparison of the cumulative incidence of EBV between Rux-Dec-mBu/Cy and historical control at 6 months (*p*=0.860). CMV, Cytomegalovirus; EBV, Epstein–Barr virus.

**Supplementary Figure 2. Outcomes of CR1, CR2, MRD-, and MRD+ after ruxolitinib combined with decitabine intensified mBu/Cy conditioning regimen.** (A) Cumulative incidence of relapse between CR1 and ≥CR2 at 2 years. (*p* < 0.001), (B) Cumulative incidence of relapse between MRD+ and MRD- at 2 years (*p*= 0.038), (C) Comparison of OS between CR1 and ≥CR2 at 2 years (*p*= 0.003), (D) Comparison of OS between MRD+ and MRD- at 2 years (*p*= 0.120); 95% CI, 95% confidence interval.

**Supplementary Figure 3. The cumulative incidence of relapse in CR1 and CR2.** (A) Comparison of the cumulative incidence of relapse between Rux-Dec-mBu/Cy and historical control at 2 years in CR1 (*p*＜0.001), (B) Comparison of the cumulative incidence of relapse between Rux-Dec-mBu/Cy and historical control at 2 years in CR2 (*p*=0.596).

**Supplementary Figure 1**

**
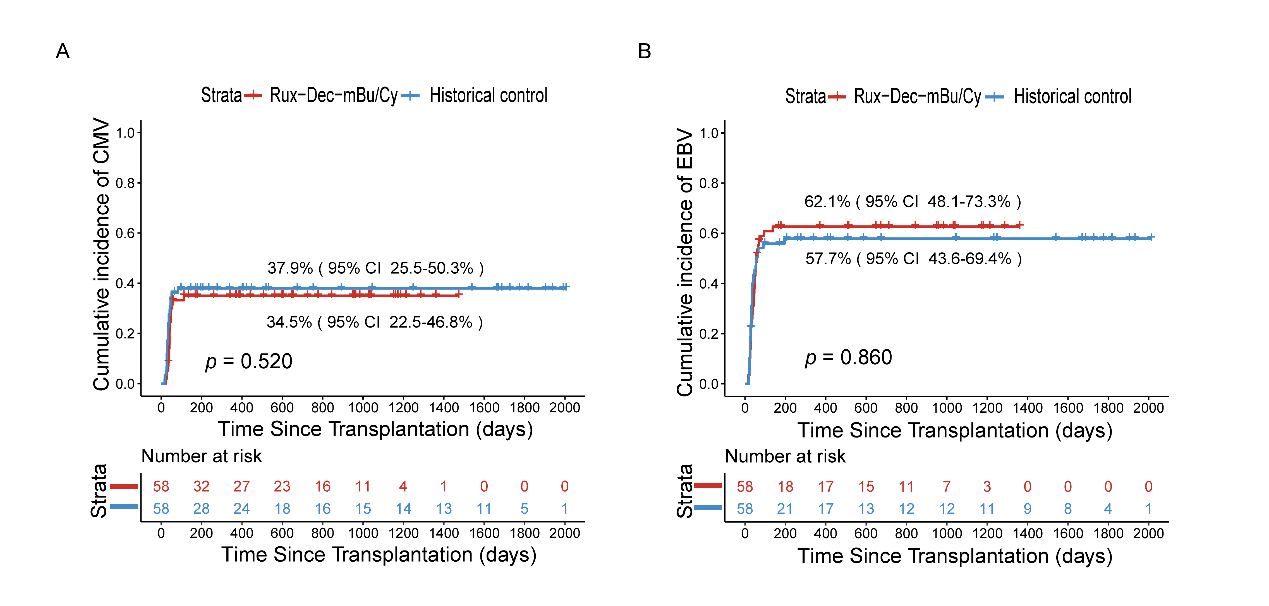
**

**Supplementary Figure 2**

**
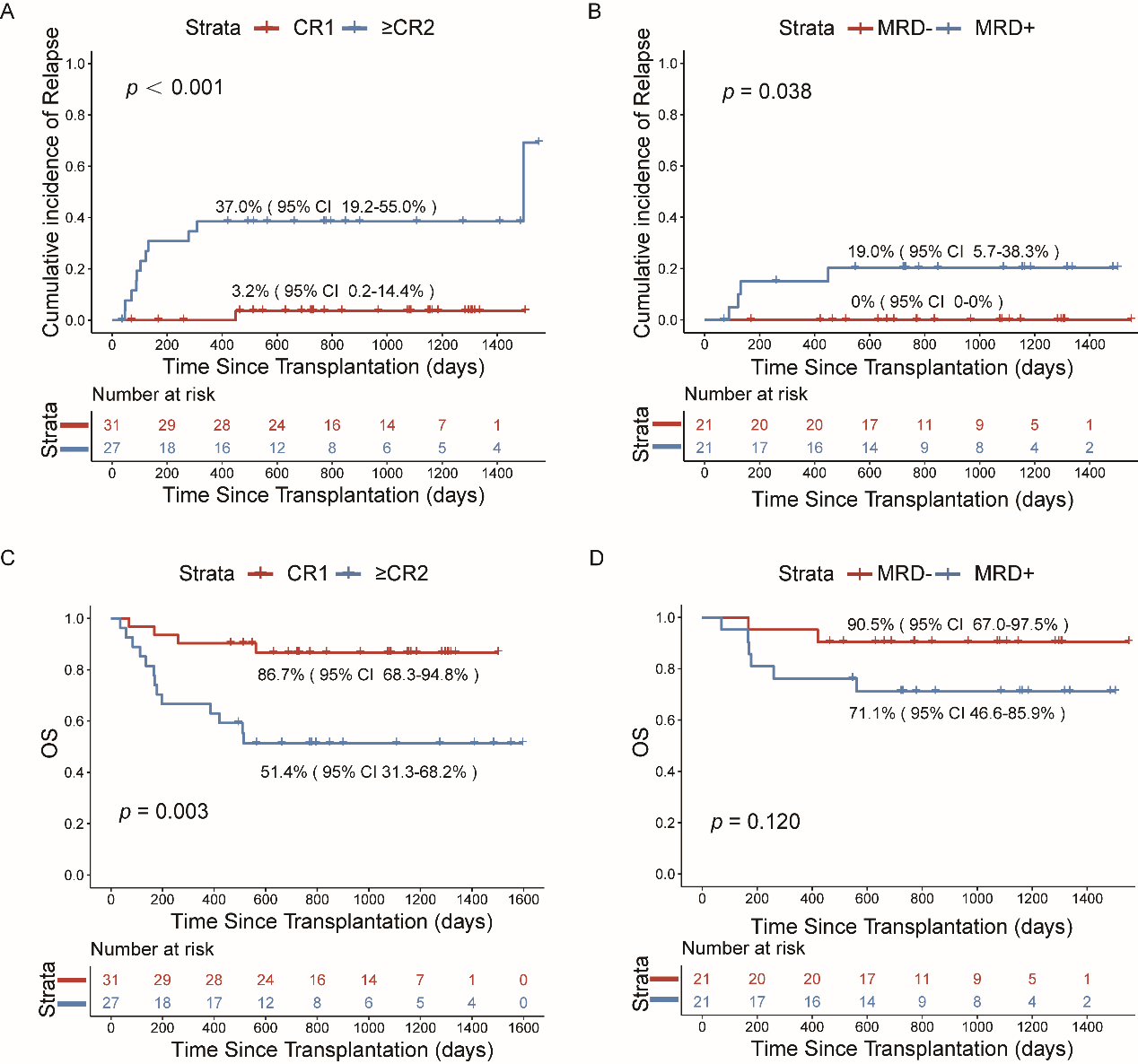
**

**Supplementary Figure 3**


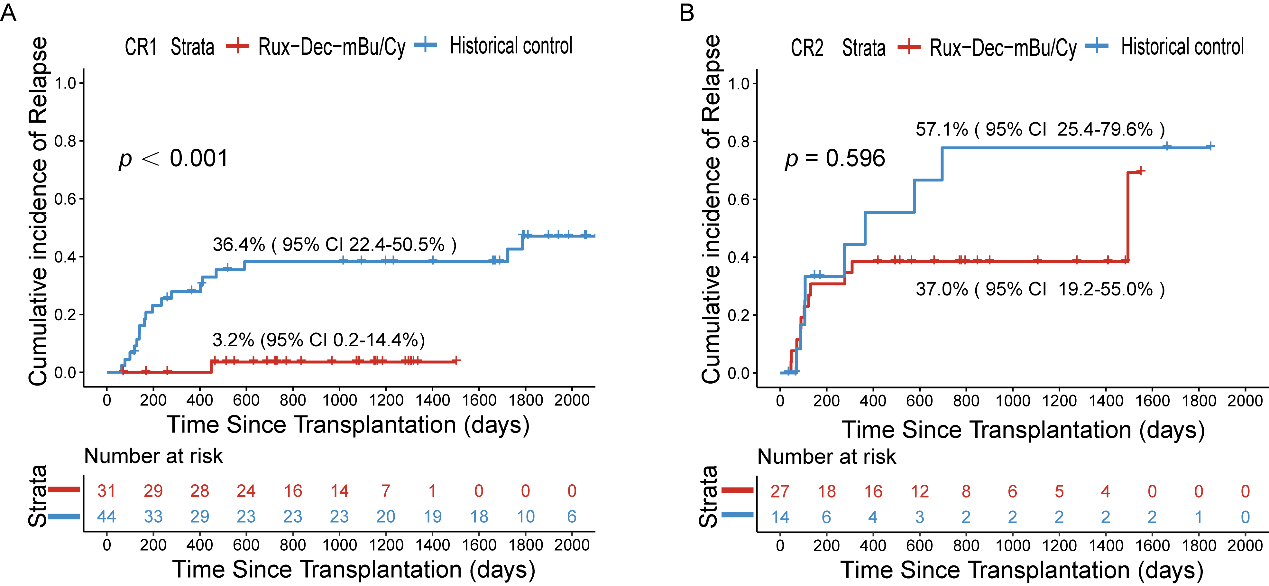

Supplement: Supplementary file 1 [file DataSheet1.docx]
